# Familial Exudative Vitreoretinopathy-Related Disease-Causing Genes and Norrin/*β*-Catenin Signal Pathway: Structure, Function, and Mutation Spectrums

**DOI:** 10.1155/2019/5782536

**Published:** 2019-11-16

**Authors:** Hongtao Xiao, Yuna Tong, Yuxuan Zhu, Min Peng

**Affiliations:** ^1^Department of Pharmacy, Sichuan Cancer Hospital & Institute, Sichuan Cancer Center, School of Medicine, University of Electronic Science and Technology of China, Chengdu, China; ^2^Personalized Drug Therapy Key Laboratory of Sichuan Province, Chengdu 610072, China; ^3^Department of Nephrology, The Third People's Hospital of Chengdu, Chengdu 610031, China; ^4^Department of Pharmacy, Sichuan Academy of Medical Sciences and Sichuan Provincial People's Hospital, School of Medicine, University of Electronic Science and Technology of China, Chengdu 610072, China; ^5^Department of Stomatology, Sichuan Academy of Medical Sciences and Sichuan Provincial People's Hospital, School of Medicine, University of Electronic Science and Technology of China, Chengdu 610072, China

## Abstract

Familial exudative vitreoretinopathy (FEVR) is a hereditary ocular disorder characterized by incomplete vascularization/abnormality of peripheral retina. Four of the identified disease-causing genes of FEVR were *NDP*, *FZD4*, *LRP5*, and *TSPAN12*, the protein coded by which were the components of the Norrin/*β*-catenin signal pathway. In this review, we summarized and discussed the spectrum of mutations involving these four genes. By the end of 2017, the number of FEVR causing mutations reported for *NDP*, *FZD4*, *LRP5*, and *TSPAN12* was, respectively, 26, 121, 58, and 40. Three most frequently reported mutations were c. 362G > A (p.R121Q) of *NDP*, c. 313A > G (p.M105V), and c.1282_1285delGACA (p.D428SfsX2) of *FZD4*. Mutations have a tendency to cluster in some “hotspots” domains which may be responsible for protein interactions.

## 1. Introduction

Familial exudative vitreoretinopathy (FEVR), described first by Criswick and Schepens in 1969 [1], is a hereditary ocular disorder characterized by incomplete vascularization/abnormality of peripheral retina. Incomplete and aberrant vascularization leads to various complications, including retinal neovascularization and exudates, retinal fold and detachments, vitreous hemorrhage, and macular ectopia, ultimately leading to total blindness.

FEVR is genetically heterogeneous and can be inherited as a dominant, recessive, or X-linked trait. The dominant form is the most common mode of inheritance. So far, mutations in at least 9 genes have been attributed to the development of FEVR including *NDP*, *FZD4*, *LRP5*, *TSPAN12*, *ZNF408*, *KIF11*, *RCBTB1*, *CTNNB1*, and *JAG1* [2–10]. The proteins encoded by the first four genes are cooperative in the Norrin/*β*-catenin signaling pathway (also named as Norrin/Frizzled-4 pathway) and showed intense interaction with each other [11]. So, this review specially focused on the mutation spectrums of these genes.

The mechanisms of *NDP*, *FZD4*, *LRP5*, and *TSPAN12* in retinal vascular had been intensively investigated during the past years. The *Ndp* knockout mouse exhibited superficial retinal vasculature development delay and was unable to form deep retinal vasculature [12]. Similarly, *FZD4* played a central role in vascular development in the eye and ear. Knockout of *Fz4* has been shown to affect vascular development both in retinal and in inner ear and cause retinal stress [13, 14]. Compared with Fzd4 or *Ndp* knockout mice, *Lrp5* knockout mice showed many milder vascular defects, in which attenuated retinal vessels and capillaries lacking lumen structure was observed [15, 16]. Afterwards, *Tspan12* was verified to cause vascular defect and affect neural cells through association with Norrin/*β*-catenin but not Wnt/*β*-catenin signaling. Formation of microaneurisms, aberrant fenestration, and delayed hyaloid vessel regression was reported in *Tspan12* knockout mice [11].

**Figure 1:**
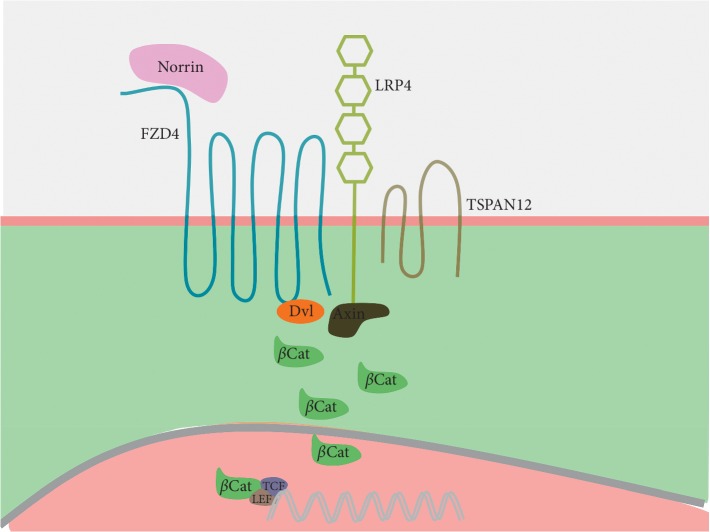
A schematic of the Norrin/*β*-catenin signal pathway. When Norrin was bond to the receptor complex FZD4/LRP5/TSPAN12, Dishevelled and Axin would be recruited to FZD5 and LRP5. Consequently, *β*-catenin escaped from the degradation complex and entered nucleus to initiate gene transcription collaborated with T-cell factor/lymphoid-enhancing factor.

In the Norrin/*β*-catenin pathway, Norrin (coded by *NDP*) worked as a ligand, while Frizzled-4 (FZD4) acted as the receptor of Norrin, in concert with low-density lipoprotein receptor-related protein-5 (LRP5) as coreceptor. Norrin binds to FZD4 and its coreceptor LRP5, forming a ternary complex. Together with the auxiliary component tetraspanin-12 (TSPAN12), this complex initiates downstream *β*-catenin signaling. Specifically, FZD4-bound Dishevelled and phosphorylated LRP5 recruited Axin to the plasma membrane, resulting in the suppression of *β*-catenin phosphorylation/degradation. The cytoplasmic levels of *β*-catenin consequently increased. Subsequently, *β*-catenin was translocated to the nucleus where it interacts with the T-cell factor/lymphoid enhancing factor, family of transcription factors, to initiate RNA transcription and elongation, as shown in Figure 1 [17–19]. This signaling pathway shared many similarities with the canonical Wnt/*β*-catenin pathway except that Norrin substituted Wnt as the ligand and traspanin-12 had been linked to the Norrin/*β*-catenin signaling pathway. Norrin/Frizzled-4 signaling plays an important role in retinal vascular growth, remodeling, and maintenance [20].

Prior to this review, a great many mutations in *NDP*, *FZD4*, *LRP5*, and *TSPAN12* had been reported by different study groups from different countries as disease-causing mutation of FEVR. Although most of the mutations were documented for once by one study group, some mutations seemed to be more common than others. Here, we presented the comprehensive list of currently known mutations in *NDP*, *FZD4*, *LRP5*, and *TSPAN12* associated with FEVR and discussed their coding consequences. This aims in facilitating the construction of a complete spectrum of mutations that occur in the above four genes. We discuss about each gene mutation individually and then highlight how they disturb the protein interactions.

## 2. Materials and Methods

The current review article aimed to analyze the studies on FEVR caused by *NDP*, *FZD4*, *LRP5*, and *TSPAN12* gene mutations to find the spectrum of these four genes. For this review study, an extensive search in PubMed and Web of Science up to December 30, 2017, was conducted independently by two individuals (Tong and Zhu) using the following search terms: “Familial exudative vitreoretinopathy” and “mutation”. To avoid losing relevant information, no limitations were set in the search. Furthermore, the related studies and the references of literatures were manually screened for additional potential eligible studies.

Mutations in NDP can result in Norrie disease and X-linked exudative vitreoretinopathy. Some earlier reports investigated Norrie disease (ND) and FEVR together. In addition, loss-of-function mutations in the LRP5 gene either cause osteoporosis pseudoglioma syndrome (OPPG) or FEVR depending on the functional severity of mutation. These distinct clinical entities share some common pathological features such as abnormal retinal blood vessel growth that may result in retinal detachment. So, we read the relevant articles of the candidates carefully to make sure the probands on whom the mutations were found were definitely diagnosed as FEVR. Then, we recorded the mutations related to FEVR and excluded those caused ND and OPPG. A total of 433 potentially relevant articles were identified, but only 41 studies involving FEVR patients caused by NDP, FZD4, LRP5, and TSPAN12 gene mutations were included in this review.

## 3. Results

### 3.1. *NDP* Mutations and Norrin Structure

The *NDP* gene locus mapped to chromosome Xp11.4 and comprised three exons. However, the first exon corresponds to the untranslated region of the gene that has regulatory functions, and only exons 2 and 3 of encode a secreted protein of 133 amino acids called Norrin or Norrie disease protein. Norrin consists of two major parts: a signal peptide at the amino-terminus of the protein that directs its localization and a region containing a typical motif of six cystines forming a cystine-knot. The cystine-knot motif is highly conserved in many growth factors as transforming growth factor-*β*, human chorionic gonadotropin, nerve growth factor, and platelet derived growth factor [21]. Cystine residues and their disulfide bonds in the cystine-knot play important structural and functional roles. Among 10 Frizzled family members, Norrin specifically binds to the transmembrane FZD4 with high affinity, forming a Norrin/FZD4 complex with LRP5 and TSPAN12 coreceptors to activate the Norrin/*β*-catenin signaling pathway [22]. Norrin was also reported to play a major role in controlling retinal vascular growth and architecture both in the developing eye and in adult vasculature.

**Figure 2:**
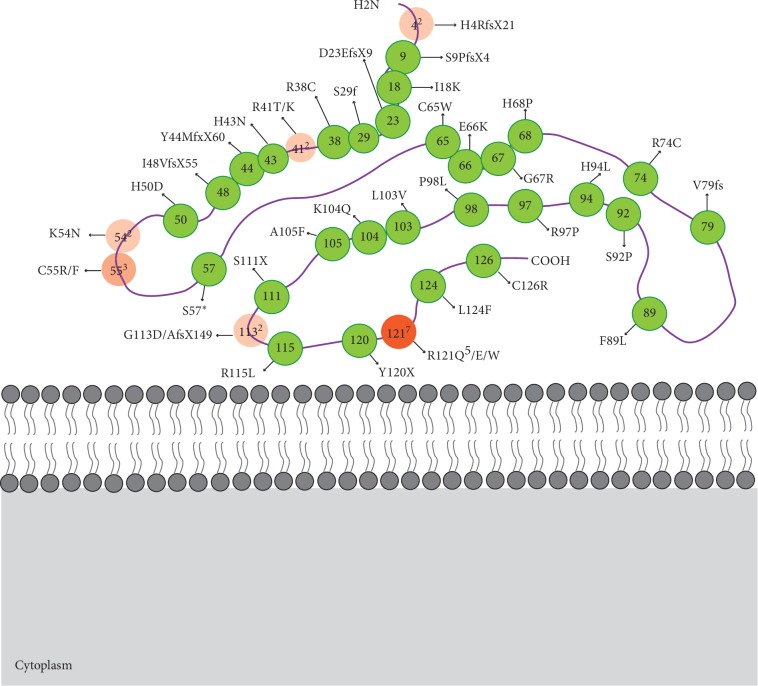
Schematic diagram of the Norrin protein shows the location of the mutations within the protein domains. Superscript number means the reported times of the same or different mutations at a certain site. The color of the mutations which were reported more than one time was recolored as orange. The opacity varied with the reported frequency of the mutations.

Twenty-six nucleotide variants have been identified for *NDP* in patients with FEVR. These include 21 missense changes, 4 deletions, and 1 insertion resulting frame shift [2, 23–31] (Table 1 and Figure 2). Most of the mutations were found in single or only a few patients, while several mutations are generally more common. By far, the most prevalent mutation was c.362G > A (p.R121Q), distributed in Spanish, Mexican, Indian, Chinese, and Italian. It is noteworthy that although probands containing c.11_12delAT (p.H4RfsX21), c.170C > G (p.S57X), and c.310A > C (p.K104Q) were definitely diagnosed as FEVR following explicit criteria, these three mutations were also reported to cause Norrie disease by other researches [28, 32, 33]. The ocular features and retinal changes observed in Norrie disease are similar to those observed in cases of FEVR. Not all the Norrie disease patients have mental retardation and develop a progressive sensorineural hearing loss; it is really difficult to distinguish Norrie disease from FEVR.

It was demonstrated from the three-dimensional structure of Norrin that two-monomer Norrins formed a homodimer in the crystal. The Norrin monomer contained exclusive *β* strands with two *β*-hairpins on one side and one *β*-hairpin on the other side. Crystal structures of Norrin in complex with the extracellular domain of FZD4 showed that two *β*-hairpins in Norrin (*β*1-*β*2 and *β*5-*β*6) interacted with three loops in FZD4 cystine-rich domain (FZD4-CRD) [38, 39]. There were 19 mutations located in domains from C39 to C65 and C96 to C126, which covered two *β*-hairpins (*β*1-*β*2 and *β*5-*β*6) and loops between them, namely, 73% of the mutations (19/26) concentrated in the interacting domains with FZD4-CRD.

Specifically, 9 mutations were located in the Norrin dimer interface which was formed from *β*2 and *β*4 sheets of one monomer and *β*2′ of another monomer (Table 1). Three mutations were reported from the cystine-knot motif, one of which (C65W) obviously impaired intermolecular disulfide bond-forming. Five mutations disturbed the hydrogen bonds or hydrophobic contacts between Norrin and FZD4 CRD in the Norrin-FZD4 CRD interface [38, 40]. Four mutations clustered on the edge of the Norrin molecule in the *β*1-*β*2 and *β*3-*β*4 loop regions were inferred as LRP5 binding sites because they did not affect *Fz4* binding yet reduced the ability of Norrin to activate the TCF reporter [39]. The residues in the interaction interface are well defined and overlap with disease-associated mutations in *NDP*. The level of signaling activity of K104Q, R121Q, and L124F was between 20% and 80% of the wide-type Norrin, suggesting that even a modest decrement in Norrin/Fz4 signaling may have a significant phenotypic effect in humans [14, 41]. It is of no surprise that the mutations located in *β*1-*β*2 and *β*5-*β*6 obstructed the formation of two *β*-hairpins and the interactions between Norrin and FZD4.

### 3.2. *FZD4* Mutations and FZD4-CRD Structure

The *FZD4* gene is located on chromosome 11q14.2, and its mRNA consists of two exons coding for 537 amino acid protein called FZD4 or Frizzled-4 protein. FZD4 acted as the receptor for Wnt and Norrin along with LRP5, which has a pivotal role in various cellular processes including cell fate determination, control of cell polarity, and malignant transformation. The FZD4 contains a ∼120-residue *N*-terminal extracellular cystine-rich domain(CRD), seven helix transmembrane domains, three extracellular and three intracellular loops, and a C terminal cytoplasmic domain [42, 43]. The cystine-rich domain is indispensable to Wnts or Norrin and is conserved among Frizzled family members [22, 39]. The FZD4 carboxyl cytoplasmic region contains juxtamembrane KTXXXW motif which is responsible for association with Dishevelled to activate downstream signaling [44, 45].

**Figure 3:**
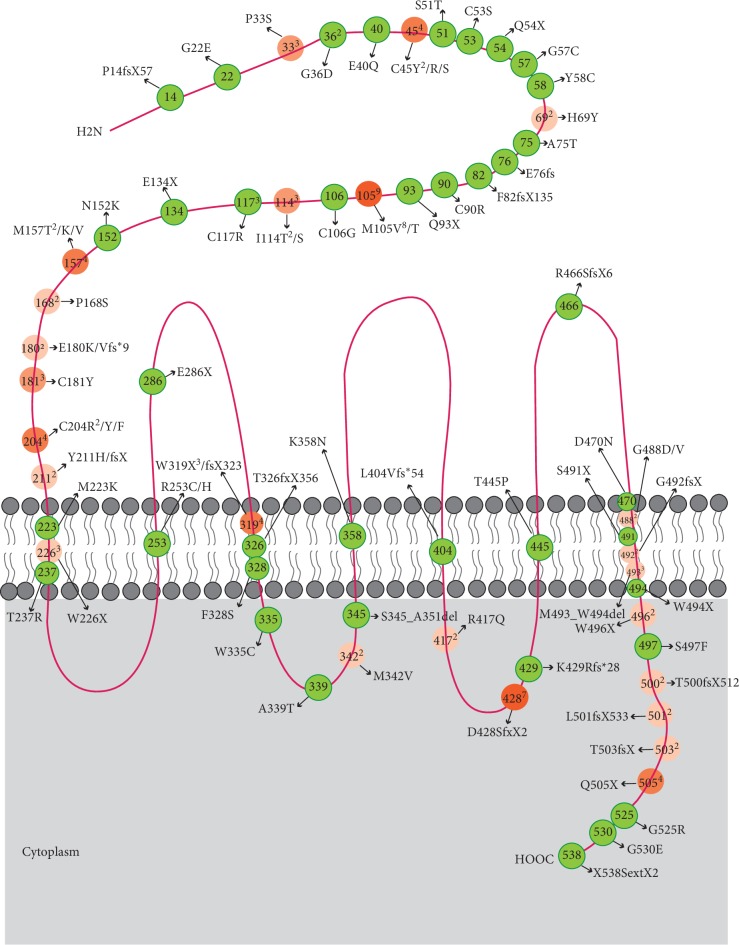
Schematic diagram of the Frizzled-4 protein shows the locations of the mutations. A whole gene deletion and a deletion/insertion (c.40 del/inser) with unknown protein change are not shown. Superscript number means the reported times of the same or different mutations at a certain site. The color of the mutations which were reported more than one time was recolored as orange. The opacity varied with the reported frequency of the mutations.

In this update, we summarized a total of 121 mutations already reported in patients with FEVR in the literatures consisting of 70 missense mutations, 19 nonsense mutations, and 30 insertions or deletions that lead to either frame shifts or in-frame deletions; a single base change resulted in 2 amino acids extension and a whole-gene deletion [7, 24, 28, 29, 46–71] (Table 2 and Figure 3). No splice mutations have been reported for *FZD4*, and the mutations seem to cluster in two specific “hotspots”. Although the mutations span in whole *FZD4* gene, 49% (59 of 121 mutations) and 13% (16 of 121 mutations) of them have a tendency to bunch in the N terminal extracellular domain and C terminal intracellular domain, respectively.

The 120-residue N-terminal extracellular cystine-rich domain (CRD) domain, connected to the first transmembrane helix by a 50-amino-acid linker, was crucial to ligand recognition. In the CRD domain, mutations at C45, M105, and M157 were three most frequently reported mutations, for 4, 9, and 4 times by different studies, respectively. One of these mutations, C45Y, was found to disrupt protein folding, resulting FZD4 being stuck in the cytoplasm with no membrane location [71]. It was supposed that the disulfide bond between Cys45 and Cys106 was imperative to protein transportation and functional activity. It was also visible from the crystal structure of FZD4-CRD that five disulfide bridges (Cys45–Cys106, Cys53–Cys99, Cys90–Cys128, Cys117–Cys158, and Cys121–Cys145) stabilized the *α* helices [38].

Two crystal structures of Norrin/FZD4-CRD complex and a FZD4 transmembrane domain had been registered in the Protein Data Bank [38, 40, 70]. The structures showed that one FZD4-CRD coupled a Norrin monomer with no interactions between the two FZD4-CRDs. Three loops between *α* helices were responsible for binding to the *β*-hairpins in Norrin [38]. The C-terminal tail of FZD4-CRD also made contribution to Norrin recognition. Residues V45, M59, L61, and L124 of Norrin and F96, M105, I110, M157, and M159 FZD4-CRD constituted a hydrophobic core at the binding interface [40]. Based on this, it is speculated that FEVR-related mutations at M105 and M157 may interrupt the binding of Norrin to FZD4. Biophysical analysis of Norrin and FZD4 demonstrated that the linker region of FZD4 contributes to a high-affinity interaction with Norrin and signaling [71]. Mutation C181Y in this domain not only destroyed the disulfide bond but also interrupted the binding of Norrin. The FZD4 transmembrane domain structure showed mutations in key positions (M309L, C450I, C507F, and S508Y) of the ΔCRD-FZD4 structure which led to aberrant downstream signaling. However, no disease-causing mutation had been reported in abovementioned four amino residuals.

The FZD4-mediated membrane recruitment of the cytoplasmic effector Dishevelled is a critical step in Wnt/*β*-catenin signaling. Considerable domains on FZD4 were identified as critical sites for recruitment of Dishevelled. A conserved motif (KTxxxW) located two amino acids after the seventh transmembrane domain was firstly verified to be crucial for membrane relocalization and phosphorylation of Dishevelled [44, 45]. The interaction between FZD4 and Dishevelled was further found to be pH- and charge-dependent [72]. Several amino residuals in intracellular loops 1, 2, and 3 and the flanking region near to intracellular loop 3 were also important for the intracellular location of Dishevelled while the mutant impaired the binding of Dishevelled [73–77]. Research based on FZD6 also showed that the linker domain, especially some conserved cystines, between the CRD domain and seven transmembrane core was imperative for Dishevelled recruitment [78]. One potential mechanism for FZD4 activation would be a Wnt/Norrin-induced movement of the seventh transmembrane domain to expose the key FZD4-Dishevelled interaction site [79]. Although 21% (26 of 121 mutations) of the mutations aggregated in the third intracellular loop and C terminal intracellular domain, it was not clear how the mutations affect the interaction between FZD4 and Dishevelled.

### 3.3. *LRP5* Mutations and LRP5/LRP6 Structure

*LRP5* gene, localized on human chromosome 11q13.2, consists of 23 exons and encodes 1615 amino acid single-pass transmembrane protein. LRP5 is a member of the low-density lipoprotein receptor family and belongs to a subfamily consisting of its mammalian homolog LRP6 and the Drosophila protein arrow. LRP5 and LRP6 share 73% identity in their extracellular domains. The LRP5/6 protein contains three domains including an extracellular domain, one transmembrane domain, and a cytoplasmic domain. The LRP5/6 ectodomain contains four *β*-propeller motifs (composed of six YWTD repeats) at the amino terminal end that alternate with four epidermal growth factor- (EGF-) like repeats (YWTD-EGF domain). These are followed by three low-density-lipoprotein receptor-like ligand-binding domains. LRP5 can act synergistically with FZD4 or other members of the Frizzled family to bind Wnts or Norrin, forming a functional ligand-receptor complex that triggers canonical Wnt/*β*-catenin or the Norrin/*β*-catenin signaling pathway and induce the transcription of target genes subsequently [80, 81].

**Figure 4:**
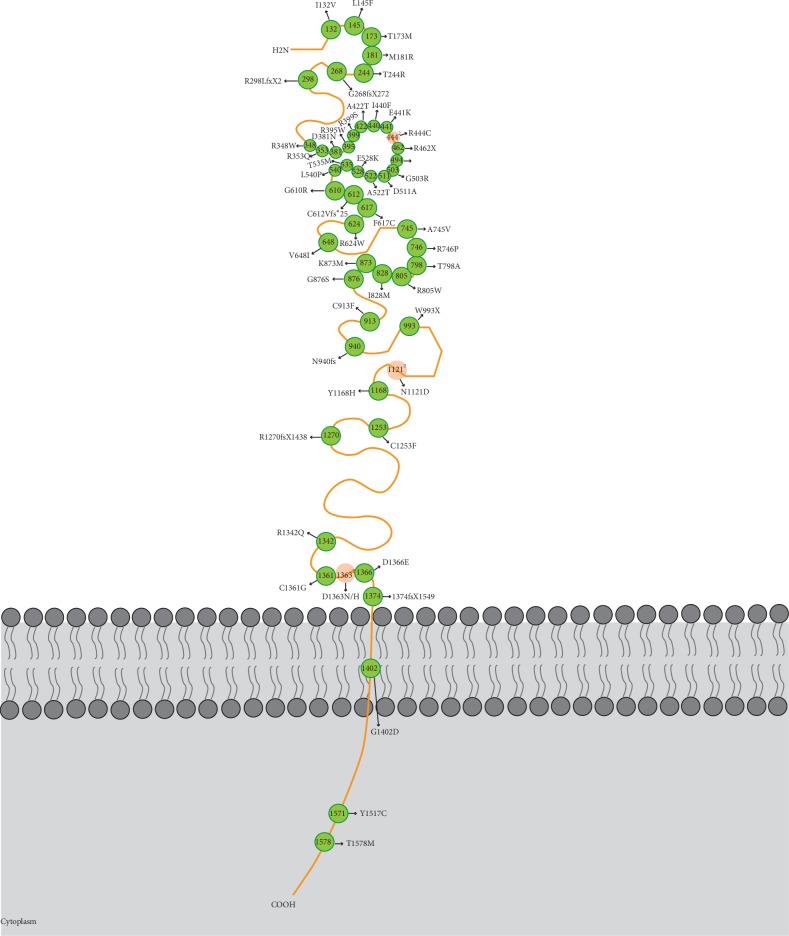
Schematic representation of LRP5 protein shows the location of the mutations within the protein domains. Four splice site mutations are not shown. Superscript number means the reported times of the same or different mutations at a certain site. The color of the mutations which were reported more than one time was recolored as orange. The opacity varied with the reported frequency of the mutations.

Thus far, 58 causative mutations identified in patients with FEVR have been reported for *LRP5*, of which 46 mutations are missense changes, 6 frame shift mutations resulted by deletions, insertion, and duplication, 2 introduce premature stop codons, and 4 changes affect splicing [28, 29, 31, 46, 56, 59, 69, 69, 82–85] (Table 3 and Figure 4). Mutations located in first, second, and third YWTD-EGF domain accounted for 12% (7 of 58 mutations), 38% (22 of 58 mutations), and 17% (10 of 58 mutations) of all the mutations, respectively. Thus, it can be seen causative mutations have a trend of clustering in the second YWTD-EGF domain since this segment is composed of only about 300 amino acids, accounting for less than 20% of whole LRP5 protein. Five of the included mutations (c.1828G>A, c.731C>G, c.1042C>T, c.1058G>A, and c.1481G>A) were also reported as causative mutation for OPPG [86], which was characterized as blindness and decreased bone density. But FEVR and OPPG were two different diseases because of the distinct pathogenesis of visual loss. OPPG patients often presented with blindness in the neonatal period and the symptoms initiated during early childhood. Inconformity of these results may was due to omission of bone density and definite pathogenesis of visual loss.

In the crystal of the first two YWTD-EGF structure of LRP6, each of the two EGF domains packs tightly against the bottom surface of the preceding YWTD *β*-propellers [87]. Extensive interface interactions was observed between the first *β*-propellers and second *β*-propellers, and the first EGF domain also interacts with the second *β*-propellers, which was critical to maintain the stability and orientation of LRP6's first two YWTD-EGF domains.

Early studies revealed that the interaction of LRP6 with Wnt-Fzd4 was mediated by the first two propeller domains [88], while other researchers pointed out that a single LRP6 might engage two different Wnt proteins simultaneously. LRP5/6 binds to different Wnts via different regions or multiple domains together [89]. The four *β*-propeller domains in LRP5/6 share a relatively low identity among them, indicating the functional differences among these YWTD propellers. Ke et al. demonstrated that Norrin interacted with *β*-propeller domain 1 (BP1) and *β*-propeller domain 2 (BP2) but not BP3-4 of LRP6. However, the binding sites of Norrin with LRP5 remain unclear. From these two perspectives, the mutations accumulated in the second YWTD-EGF domain may destroy the stable structure of first two *β*-propellers or interrupted their interaction with Norrin or Fzd4.

### 3.4. *TSPAN12* Gene, Protein, and Spectrum

The *TSPAN12* gene is located on chromosome 7q31 and encodes for a 305 amino acid transmembrane protein. TSPAN12 is a member of the tetraspanin family that shares certain specific structural features that distinguishes them from other proteins that pass the membrane four times. Both the N and C terminals of TSPAN12 were inside the cell membrane, and it has an unusually long C-terminal intracellular tail of approximately 60 amino acids. It contains four transmembrane domains connected by two extracellular loops (ECL-1 and ECL-2) and an intracellular loop. The ECL-1 is smaller compared to the ECL-2.

TSPAN12 was discovered to associate selectively with Norrin/*β*-catenin signaling but not with Wnt/*β*-catenin signaling. It acted as the fourth important component of Norrin/FZD4/LRP5 complex. Signaling reduction could be rescued by TSPAN12 overexpression although direct binding with Norrin and FZD4 was not detected. However, another study reported that TSPAN12 interacted with Norrin and FZD4 via its extracellular loops and enhanced the FZD4 ligand selectivity for NDP [90]. Thus, TSPAN12 was postulated to elicit physiological levels of signaling that was required for normal retinal angiogenesis by promoting FZD4 multimerization cooperated with Norrin and facilitating selective ligand recognition [11].

**Figure 5:**
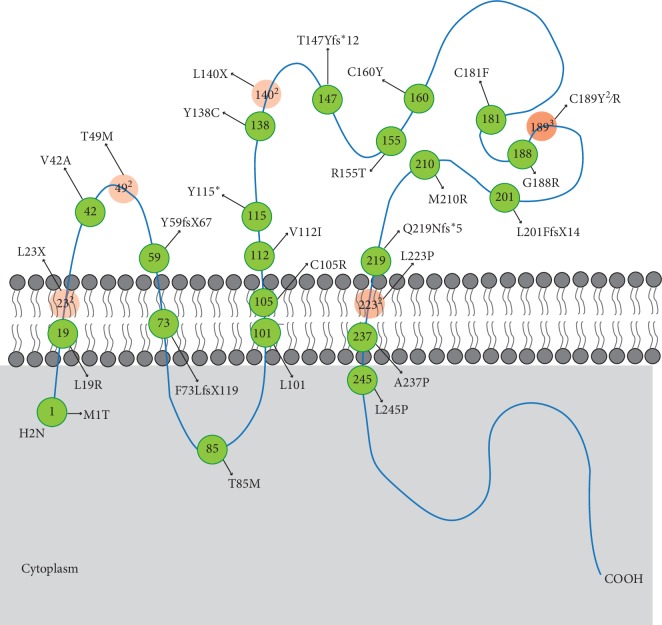
Schematic diagram of the TSPAN12 protein shows the location of the 31 known mutations within the protein domains. Nine splice-site mutations besides one mutation (c.916-918 + 3delTAAAAA) resulting in protein extension and one mutation (c.67-1G>C) resulting in frame shift are not shown in this diagram. Superscript number means the reported times of the same or different mutations at a certain site. The color of the mutations which were reported more than one time was recolored as orange. The opacity varied with the reported frequency of the mutations.

We summarized 40 currently known mutations in TSPAN12 identified in patients affected with FEVR and discussed their coding consequences [6, 24, 29, 31, 54, 58, 59, 68, 83, 91–96] (Table 4 and Figure 5). All types of mutations were identified, including 22 missense mutation, 4 nonsense mutations, 9 splice-site mutations, 3 deletions, and 2 insertions. Mutations at residues T49, L140, C189, and L233 were reported more than one time. It was reported that L233P strongly impaired the TSPAN12 activity, while T49M mildly impaired the activity. Unfortunately, the authors did not investigate the signaling defect strength of L140X and C189Y/R. In all of the mutations, 38% (15 in 40 mutations) of them were located in the ECL-2 domain. These mutations were highly consistent with the biochemical results. TSPAN12 is anchored to the Norrin receptor complex via an interaction of the LEL with FZD4. The ECL-2 domain of TSPAN12 is essential for enhancing Norrin-induced FZD4 signaling. TSPAN12 can also alleviate the defects of FZD4 M105V, a mutation that destabilizes the NDP/FZD4 interaction [90].

## 4. Discussion

FEVR causing *NDP*, *FZD4*, *LRP5*, and *TSPAN12* mutations was reported from 15 countries including USA, UK, China, Spain, India, Australia, Mexico, Japan, Netherlands, Italy, Canada, Korea, Sweden, Pakistan, and Israel. Top three countries with the largest number of reported mutations about *NDP*, *FZD4*, *LRP5*, and *TSPAN12* genes were China, Netherlands, and Japan. The number of reported mutations did not completely match the population, since the three most populous countries were China, India, and USA. One of the major reasons contributing to this phenomenon might be the number of research groups was more in China, Netherlands, and Japan than that in other regions. Although most of the mutations were reported by only one study just once, some specific mutations were more common than others. For example, mutations of *NDP* at c.362G (p.R121) was independently reported by 5 different studies and distributed in Spanish, Indian, Mexican, Chinese, and Italian. *FZD4* c. 313A>G (p.M105V) was reported for 8 times by 8 different research groups. Thus, it is significant to investigate the structure and function changes of the coding protein which resulted by the widely reported mutations.

Although the mutations scattered widely through the whole genes, they have an inclination to distribute in certain areas. From the point of view of the coding proteins, the mutations concentrated at the N-terminal and C-terminal domains of Norrin. There were 19 mutations located in domains from C39 to C65 and C96 to C126, which covered the two *β*-hairpins (*β*1-*β*2 and *β*5-*β*6) and loops between and was crucial for binding with FZD4-CRD, namely, 73% of the mutations (19/26) concentrated in the interacting domains with FZD4-CRD. In terms of *FZD4*, 49% (59 of 121 mutations) of the mutations were positioned in the extracellular domain, which played a significant role in ligand recognition, while 13% (16 of 121 mutations) of the mutations were positioned in the intracellular domain which recruited Dishevelled to activate downstream signaling. The sum of mutations from the two domains accounted for 61% of total reported mutations. The tendency of mutations accumulating in certain domains was more obvious in regard to LRP5 protein. More than a third of reported mutations (38%, 22/58) were found from the second YWTD-type *β*-propeller domain and EGF domain, which were comprised of approximately 300 amino acids, accounting for less than 20% of whole LRP5 protein. But whether the second YWTD-EGF domains interacted with Norrin and FZD4 directly or not remained unknown. As far as TSPAN12 was concerned, it seemed that the mutations were intensively located in the ECL-2 domain (38%, 15/40). A recent study revealed that the large extracellular loop of TSPAN12 is required for enhancing Norrin-induced FZD4 signaling. In conclusion, the “hotspots” where mutations clustered were highly consistent with the domains participating protein interactions.

Overall, mutations in *NDP*, *FZD4*, *LRP5*, and *TSPAN12* genes explained up to ∼50% of all FEVR cases worldwide [97]. Besides the four genes we reviewed in this review, *ZNF408*, *KIF11*, *RCBTB1*, *CTNNB1*, and *JAG1* were also reported to be the disease-causing genes of FEVR. The proteins encoded by *NDP*, *FZD4*, *LRP5*, *TSPAN12*, and *CTNNB1* genes participate in the Norrin/*β*-catenin pathway, the signaling which is critical for retinal angiogenesis by controlling retinal vascular growth and architecture. The connection of proteins coded by *ZNF408*, *KIF11*, and *RCBTB1* genes with the Norrin/*β*-catenin pathway was still unclear. A comprehensive spectrum covering other four causative genes (*ZNF408*, *KIF11*, *RCBTB1*, and *CTNNB1*) and further investigation on the biochemical functions of their coding proteins will undoubtedly facilitate thorough understanding of the pathogenic mechanism of FEVR.

Pathogenic mutations in *NDP* and *FZD4* lead to a number of retina-related diseases including FEVR, Norrie disease, persistent hyperplastic primary vitreous, advanced stage of retinopathy of prematurity, and Coats disease. These diseases can be diagnosed according to their unique symptoms which can be distinguished from FEVR [98]. The common characteristic of these *NDP* and *FZD4* related diseases was defects in the vascularization of the retina. Further study on the role of the Norrin/*β*-catenin pathway in the retinal vascular may promote the understanding of the mechanism of the pathogenic mutations [12]. Furthermore, other sprouting angiogenesis associated components will in some way help provide in-depth insight about these retina-related diseases.

## Figures and Tables

**Table 1:** Spectrum of *NDP* gene mutations among patients with familial exudative vitreoretinopathy.

Studies	No. of patients	No. of mutations	DNA variant	Coding effect	Location of the amino residue	Mutant phenotypes	Country of origin
Chen et al. [2]	30	1	c.370C>T	p.L124F	Norrin dimer interface	Retina detached	UK
Riveiro-Alvarez et al. [30]	45	1	c.362G>A	p.R121Q	Norrin dimer interface	Congenital blindness, phthisis bulbi	Spain
Dickinson et al. [23]	13	1	c.307C>G	p.L103V	Norrin-FZD4 interface	Not mentioned	Australia
Hiroyuki et al. [34]	62	3	c.53T>A	p.I18K	Signal domain	Peripheral avascularization, neovascularization	Japan
			c.162G>C	p.K54N	Deductive Norrin-LRP5 interface	Retinal detachment and macular traction with temporal avascularization	
			c.344G>T	p.R115L	Deductive Norrin-LRP5 interface	Retinal detachment	
Pelcastre et al. [35]	127	3	c.361C>T	p.R121W	Norrin dimer interface	On-perfusion in peripheral retina	Mexico
			c.362G>A	p.R121Q	Norrin dimer interface	Retinal detachment	
Musada et al. [36]	110	8	c.11_12delAT	p.H4RfsX21	Signal domain	Bilateral total retinal detachment	India
			c.69delC	p.D23EfsX9	Signal domain	Pigmentation and vitreoretinal traction	
			c.142_145delATCA	p.I48VfsX55	Premature termination	Bilateral leukocoria and total retinal detachment	
			c.148C>G	p.H50D	Deductive Norrin-LRP5 interface	Straightening of the blood vessel, macular dragging	
			c.170C>G	p.S57X	Norrin-FZD4 interface	Retinal detachments and retrolental membranes	
			c.338G>A	p.G113D	Near deductive Norrin-LRP5 interface	Avascular peripheral retina, straightening of the blood vessels, and dye leakage	
			c.362G>A	p.R121Q	Norrin dimer interface	Retinal detachments with retrolental membranes	
			c.376T>C	p.C126R	Norrin dimer interface	Bilateral total retinal detachment	
Liu Y. L. et al. [37]	40	1	c.310A>C	p.K104Q	Norrin-FZD4 interface	Weak eyesight, retinal vascular abnormalities	China
Tang et al. [31]	100	5	c.196G>A	p.E66K	Cystine-knot motif	Macular dragging	China
			c.203A>C	p.H68P	Cystine-knot motif	Ectopic macular	
			c.281A>T	p.H94L	Norrin dimer interface	Peripheral avascular zone and retinal exudates	
			c.362G>A	p.R121Q	Norrin dimer interface	Retinal fold, retinal detachment	
			c.334delG	p.G113AfsX149	Premature termination	Bilateral tractional retinal detachment	
Iarossi et al. [24]	8	2	c.362G>A	p.R121Q	Norrin dimer interface	Falciform fold, partial traction	Italian
			c.313G>C	p.A105F	Norrin-FZD4 interface	Macula-involving retinal detachment	
Rao et al. [29]	31	3	c.127C>A	p.H43N	Norrin-FZD4 interface	Complete retinal detachment	China
			c.52_53ins32bp	p.S29fs	Premature termination	Complete retinal detachment	
			c.195C>G	p.C65W	Cystine-knot motif, form disulfide bond with C126	Complete retinal detachment	

**Table 2:** Spectrum of *FZD4* gene mutations among patients with familial exudative vitreoretinopathy.

Studies	No. of patients	No. of mutations	DNA variant	Coding effect	Location of the amino residue	Mutant phenotype	Country of origin
Zhang et al. [65]	49	5	c.134G>A	p.C45Y	CRD domain, no plasma membrane localization, failed to mediate Norrin induction of these *β*-catenin target genes	Not mentioned	China and USA
			c.173A>G	p.Y58C	CRD domain, failed to bind Norrin, failed to mediate Norrin induction of these *β*-catenin target genes	Not mentioned	
			c.610T>C	p.C204R	CRD domain, failed to bind Norrin, failed to mediate Norrin induction of these *β*-catenin target genes	Not mentioned	
			c.678G>A	p.W226X	Transmembrane 1, failed to mediate Norrin induction of these *β*-catenin target genes	Not mentioned	
			c.1488G>A	p.W496X	C-terminal intracellular domain, failed to mediate Norrin induction of these *β*-catenin target genes	Not mentioned	

Drenser et al. [48]	123	5	c.97C>T	p.P33S	Signal sequence	2-stage FEVR, rhegmatogenous retinal detachment	USA
			c.349T>C	p.C117R	CRD domain, conserved cystine residue	4B stage FEVR	
			c.502C>T	p.P168S	CRD domain	2-stage FEVR, rhegmatogenous retinal detachment	
			c.542G>A	p.C181Y	CRD domain, conserved cystine residue	4B stage FEVR	
			c.1513C>T	p.Q505X	Immediately downstream from KTxxxW motif	4B stage FEVR	

Qin et al. [56]	56	2	c.1005G>C	p.W335C	Highly conserved across all members of the FZD family	Bilateral retinal folds	Japan
			c.1024A>G	p.M342V	Intracellular loop 2, function not shown	Bilateral dragged disc	

Robitaille et al. [7]	27	2	c.1479_1484del	p.M493_W494del	Failed to activate calcium/calmodulin-dependent protein kinase II and protein kinase C	Bilateral retinal detachment	Canada
			c.1501_1502delCT	p.L501fsX533	No membrane accumulation, failed to activate calcium/calmodulin-dependent protein kinase II and protein kinase C	Not mentioned	

Kondo et al. [51]	24	4	c.313A>G	p.M105V	CRD domain	Bilateral vitreous opacity, retinal exudates, macular ectopia, falciform retinal fold	Japan
			c.957G>A	p.W319X	Transmembrane domain	Falciform retinal fold, chronic retinal detachment	
			c.1250G>A	p.R417Q	Intracellular loop 3	Falciform retinal fold, posterior synechiae, chronic retinal detachment	
			c.1463G>A	p.G488D	Transmembrane domain	Falciform retinal folds	
Dailey et al. [47]	421	11	c.40 Del/inser	Unknown	Not mentioned	Not mentioned	USA
			c.97C>T	p.P33S	Signal sequence, reduced Wnt reporter activity	Not mentioned	
			c.151T>A	p.S51T	CRD domain	Not mentioned	
			c.169G>T	p.G57C	CRD domain	Not mentioned	
			c.349T>C	p.C117R	CRD domain	Not mentioned	
			c.502C>T	p.P168S	CRD domain, reduced Wnt reporter activity	Not mentioned	
			c.542G>A	p.C181Y	CRD domain	Not mentioned	
			c.758G>A	p.R253H	Transmembrane domain	Not mentioned	
			c.1074A>C	p.K358N	Transmembrane domain	Not mentioned	
			c.1513C>T	p.Q505X	Immediately downstream from KTxxxW motif	Not mentioned	
			c.1589G>A	p.G530E	C-terminal	Not mentioned	

Fei et al. [49]	61	3	c.C205T	p.H69Y	CRD domain	Not mentioned	China
			c.G400T	p.E134X	CRD domain, failed to activate *β*-catenin reporter	Peripheral avascular zone, dragged disc	
			c.1506delAC	p.T503fs	Failed to activate *β*-catenin reporter	Total retinal detachment	

Yang et al. [69]	56	5	c.313A>G	p.M105V	CRD domain	Increased branching of peripheral vessels, retinal detachment, Avascular zone, Retrolenticular fibrotic mass, neovascularization	China
			c.631T>C	p.Y211H	Linker upstream of transmembrane 1	Temporal dragging of optic disc, peripheral fibrous proliferation	
			c.1282-1285delGACA	p.D428SfsX2	Intracellular loop 3	Straightening of temporal arcades, temporal dragging of optic disc, peripheral fibrous proliferation	
			c.1482G>A	p.W494X	Transmembrane domain	Retrolenticular fibrotic mass, lens dislocation, brushlike peripheral, avascular zone, neovascularization, peripheral fibrous proliferation	
			c.1513C>T	p.Q505X	Immediately downstream from KTxxxW motif	Temporal dragging of optic disc, f alciform retinal fold, branching of peripheral vessels, avascular zone, peripheral exudates	

Nallathambi et al. [55]	75	3	c.97C>T	p.P33S	Signal sequence, reduced Wnt reporter activity	Peripheral lattice degeneration, atrophic holes, macular ectopia, bilateral peripheral avascular zone	India
			c.244_251del8ins27	p.F82fsX135	CRD domain	Macular ectopia, terminal branching, peripheral avascular zone	
			c.610T>C	p.C204R	CRD domain	Temporal peripheral avascular zone, terminal branching, tractional retinal detachment	
Seo et al. [59]	51	9	c.160C>T	p.Q54X	CRD domain	1B stage FEVR	Korea
			c.313A>G	p.M105V	CRD domain	1B,1A, 2B stage FEVR	
			c.456C>G	p.N152K	CRD domain	1B, 2B stage FEVR	
			c.470T>C	p.M157T	CRD domain	1B stage FEVR	
			c.539_540delAG	p.E180VfsX9	CRD domain	1B, 3A stage FEVR	
			c.676T>A	p.W226R	Linker upstream of transmembrane 1	1B, 3A stage FEVR	
			c.1210_1211delTT	p.L404VfsX54	Transmembrane domain	1A stage FEVR	
			c.1282_1285delGACA	p.D428SfsX2	Intracellular loop 3	1A stage FEVR	
			Whole gene deletion	No protein	No protein	2B stage FEVR	

Musada et al. [66]	110	7	c.313A>G	p.M105V	CRD domain	Diagnosed with FEVR, symptoms not mentioned	Indian
			c.341T>G	p.I114S	CRD domain	Diagnosed with FEVR, symptoms not mentioned	
			c.470T>C	p.M157T	CRD domain	Diagnosed with FEVR, symptoms not mentioned	
			c.1282_1285delGACA	p.D428SfsX2	Intracellular loop 3	Diagnosed with FEVR, symptoms not mentioned	
			c.1286_1290delAGTTA	p.K429RfsX28	Intracellular loop 3	Diagnosed with FEVR, symptoms not mentioned	
			c.1395_1396insT	p.R466SfsX6	Extracellular loop	Diagnosed with FEVR, symptoms not mentioned	
			c.1613A>C	p.X538SextX2	C-terminus	Diagnosed with FEVR, symptoms not mentioned	

Jia et al. [50]	48	12	c.39-49delCCCGGGGGCG	p.P14fsX57	Signal sequence, Truncated protein	Avascular retina, dragged macula	China
			c.65G>A	p.G22E	Signal sequence, loss of activity	Nystagmus, retrolental fibroplasia, retinal detachment	
			c.205C>T	p.H69Y	CRD domain, loss of activity	Avascular retina, fibrous proliferation, and dragged macula	
			c.313A>G	p.M105V	CRD domain, loss of activity	Retinal vascular tortuosity, exudates, and avascularization	
			c.538G>A	p.E180K	CRD domain, loss of activity	Not mentioned	
			c.710C>G	p.T237R	Linker upstream of transmembrane 1, loss of activity	Preretinal fibrosis, peripheral nonperfusion	
			c.757C>T	p.R253C	Transmembrane domain, loss of activity	Peripheral avascularization and typical scalloped border	
			c.983T>C	p.F328S	Transmembrane domain, loss of activity	Retinal folds and persistent hyperplastic primary vitreous	
			c.1015G>A	p.A339T	Intracellular loop 2, loss of activity	Dragged discs, retinal elevation with hemorrhage	
			c.1408G>A	p.D470N	Transmembrane domain, loss of activity	Dragged discs, retinal folds, and macular ectopia	
			c.1472C>A	p.S491X	Transmembrane domain, truncated protein	Dragged discs, macular ectopia, and pigment changes	
			c.1488G>A	p.W496X	C-terminus, truncated protein	Not mentioned	

Peachey et al. [70]	1	1	c.1026A>G	p.M342V	Intracellular loop 2	Straightening of the retinal vessels, peripheral avascular areas	Japanese
Tang et al. [60]	100	14	c.107G>A	p.G36D	Signal sequence	Not mentioned	China
			c.133T>C	p.C45R	CRD domain	Avascular zone, increasing of peripheral vessels, straightening of vessels	
			c.133T>A	p.C45S	CRD domain	Not mentioned	
			c.134G>A	p.C45Y	CRD domain	Not mentioned	
			c.158G>C	p.C53S	CRD domain	Macular dragging, Avascular zone, increasing of peripheral vessels, straightening of vessels	
			c.223G>A	p.A75T	CRD domain	Not mentioned	
			c.268T>C	p.C90R	CRD domain	Not mentioned	
			c.313A>G	p.M105V	CRD domain	Not mentioned	
			c.957G>A	p.W319X	Transmembrane domain	Avascular zone, increasing of peripheral vessels	
			c.975_978delCACT	p.T326fsX356	Transmembrane domain	Avascular zone, neovascularization, increasing of peripheral vessels, SV	
			c.1034_1054delCTTATTTCCACATTGCAGCCT	p.S345_A351del	Intracellular loop 2	Avascular zone, increasing of peripheral vessels, straightening of vessels	
			c.1282_1285delGACA	p.D428SfsX2	Intracellular loop 3	Not mentioned	
			c.1475delG	p.G492fsX512	Intracellular loop 3	Avascular zone, neovascularization, increasing of peripheral vessels, straightening of vessels,vessels exudates	
			c.1498delA	p.T500fsX512	Truncated protein	Not mentioned	

Nikopoulos et al. [68]	16	5	c.118G>C	p.E40Q	Signal sequence	Not mentioned	Netherlands
			c.611G>A	p.C204Y	CRD domain	Deformation of posterior retina, ectopia of the macula, stretched retinal vessels, retinal detachment	
			c.856G>T	p.E286X	Extracellular loop	Few abnormal temporal retinal branches, avascular peripheral fundus	
			c.1282_1285del	p.D428SfsX2	Intracellular loop 3	Macular ectopia. Haemorrhagic and exudative areas present in the retina	
			c.1573G>C	p.G525R	C-terminus	Macular ectopia and peripheral, retinal detachment	

Kondo et al. [25]	1	1	c.1250G>A	p.R417Q	Intracellular loop 3	retrolental fibroplasia, falciform retinal fold	Japanese
Toomes et al. [69]	40	8	c.107G>A	p.G36D	Signal sequence	Unable to obtain detailed clinical notes	UK
			c.314T>C	p.M105T	CRD domain	Macula-off rhegmatogenous retinal detachment, inadequate vascularization	
			c.469A>G	p.M157V	CRD domain	Macular folds and retinal detachments	
			c.957delG	p.W319fsX323	Transmembrane domain	Peripheral retinal fold	
			c.1490C>T	p.S497F	C-terminus	Disc-dragging	
			c.1498delA	p.T500fsX512	KTxxxW domain	Small myopic optic disc, diffuse nonspecific pigmentary changes	
			c.1501_1502delCT	p.L501fsX533	KTxxxW domain	Bilateral cicatrized tractional retinal detachments	
			c.1513C>T	p.Q505X	Immediately downstream from KTxxxW motif	Temporal sector of retina with deficient vascularization	

Robitaille et al. [57]	68	11	c.316 T>C	p.C106G	CRD domain	Dragging of the retina, macular fold	Canadian
			c.470T>A	p.M157K	CRD domain	Peripheral pigmentary, total retinal detachment, nonperfusion with leucocoria	
			c.633delC	p.Y211fsX	Linker upstream of transmembrane 1	Haemangiomatous lesion with exudation and peripheral avascular retina	
			c.1282_1285del	p.D428SfsX2	Intracellular loop 3	Left macula dragged	
			c.1463G>T	p.G488V	Transmembrane domain		
			c.1508insC	p.T503fsX31	KTxxxW motif	Bilateral dragging of the macula with peripherally straightened, avascular retina	
			c.313A>G	p.M105V	CRD domain	Bilateral dragging of the macula, retina detachment	
			c.678G>A	p.W226X	Linker upstream of transmembrane 1	Large elevated tight fold, large falciform fold	
			c.1448G>A	p.W496X	C-terminal intracellular domain	Tractional retinal detachment	
			c.1479_1484del	p.M493_W494del	Transmembrane domain	Not mentioned	
			c.341T>C	p.I114T	CRD domain	Not mentioned	
Robitaille et al. [67]	5	2	c.1479_1484del	p.M493_W494del	Transmembrane domain	Absence of retinal vasculature, hypoplastic iris with posterior synechiae	Canadian
			c.341T>C	p.I114T	CRD domain	Falciform retinal folds, small atrophic retinal hole	

Boonstra et al. [46]	83	4	c.668T>A	p.M223K	Linker upstream of transmembrane 1	Diagnosed with FEVR, symptoms not mentioned	Netherlands
			c.957G>A	p.W319X	Transmembrane domain	Diagnosed with FEVR, symptoms not mentioned	
			c.1333A>C	p.T445P	Transmembrane domain	Diagnosed with FEVR, symptoms not mentioned	
			c.1448G>A	p.W496X	C-terminus, truncated protein	Diagnosed with FEVR, symptoms not mentioned	

Iarossi et al. [24]	8	3	c.277C>T	p.Q93X	CRD domain	Large avascular area, falciform retinal fold	Italian
			c.542G>A	p.C181Y	CRD domain	Stage 3 and stage 2 FEVR	
			c.611G>T	p.C204F	CRD domain	Stage 4A FEVR	

Rao et al. [29]	31	2	c.1282_1285delGACA	p.D428SfsX2	Intracellular loop 3	Complete retinal detachment	China
			c.227delA	p.E76fs	CRD domain	Falciform retinal detachment.	

Murken et al. [53]	1	1	c.1474delG	p.G492fsX	Intracellular loop 3	Peripheral avascular zone and macular dragging	Mexico

Schatz and Khan [58]	3	1	c.349T>C	p.C117R	CRD domain, forms a disulfide bond with Cys158	Mild temporal avascularity, mild peripheral temporal avascularity	Sweden

**Table 3:** Spectrum of *LRP5* gene mutations among patients with familial exudative vitreoretinopathy.

Studies	No. of patients	No. of mutations	DNA variant	Coding effect	Location of the amino residue	Mutant phenotype	Country of origin
Toomes et al. [68]	32	6	c.518C>T	p.T173M	First *β*-propeller motif	Abnormal retinal vasculature and retinal fold	USA
			c.3502T>C	p.Y1168H	Low-density-lipoprotein receptor-like ligand binding domains	Total retinal detachment and retinoschisis	
			c.3840delA	p.R1270fsX1438	Premature termination	Not mentioned	
			c.4081T>G	p.C1361G	Low-density-lipoprotein receptor-like ligand binding domains	Classic features of FEVR	
			c.4119_4120insC	p.K1374fsX1549	Premature termination	Not mentioned	
			c.4488 + 2T>G	Splice-donor mutation	Premature termination	Undetermined	

Qin et al. [56]	56	9	c.433C>T	p.L145F	First *β*-propeller motif	Bilateral retrolental fibroplasias and total retinal detachment	Japan
			c.803_812del	p.G268fsX272	Premature termination	Bilateral dragged macula	
			c.1330C>T	p.R444C	Second *β*-propeller motif	Severe falciform retinal fold	
			c.1564G>A	p.A522T	Second *β*-propeller motif	Tractional retinal detachment, severe macular ectopia along with peripheral fibrovascular mass	
			c.1604C>T	p.T535M	Second *β*-propeller motif	Bilateral retinal folds followed by total retinal detachment	
			c.1828G>A	p.G610R	Second epidermal growth-like factor	Bilateral dragged macula	
			c.1850T>G	p.F617C	Second epidermal growth-like factor	Bilateral retinal folds followed by total retinal detachment	
			c.2392A>G	p.T798A	Third *β*-propeller motif	Bilateral peripheral avascular retinas	
			c.3361A>G	p.N1121D	Fourth *β*-propeller motif	Unilateral falciform retinal fold with bilateral retinal avascularization	

Boonstra et al. [46]	83	2	c.1532A>C	p.D511A	Second *β*-propeller motif	Diagnosed with FEVR, symptoms not mentioned	Netherlands
			c.2413C>T	p.R805W	Third *β*-propeller motif	Diagnosed with FEVR, symptoms not mentioned	

Nikopoulos et al. [28]	16	4	c.1321G>A	p.E441K	Second *β*-propeller motif	Not mentioned	Netherlands
			c.2978G>A	p.W993X	EGF-like domain following the third “*β*-propeller” module	Not mentioned	
			c.3758G>T	p.C1253F	EGF-like domain following the third “*β*-propeller” module	Not mentioned	
			c.4489-1G>A	Splice defect	Not applicated	Not mentioned	

Yang et al. [69]	49	6	c.891-892delTC	p.R298LfxX2	Premature termination	Retrolenticular fibrotic mass, retinal detachment, microcornea, flat anterior chamber	China
			c.2484C>G	p.I828M	Third *β*-propeller motif	Retrolenticular fibrotic mass, stretched ciliary process	
			c.2626G>A	p.G876S	Third epidermal growth like factor	Retrolenticular fibrotic mass, stretched ciliary process	
			c.3361A>G	p.N1121D	Fourth *β*-propeller motif	Temporal dragging of optic disc, retrolenticular fibrotic mass	
			c.4025G>A	p.R1342Q	Low-density-lipoprotein receptor-like ligand binding domains	Microcornea, retrolenticular fibrotic mass, avascular zone	
			c.4087G>A	p.D1363N	Low-density-lipoprotein receptor-like ligand binding domains	Increased branching of peripheral vessels, retrolenticular fibrotic mass	

Fei et al. [82]	2	2	c.1264G>A	p.A422T	Second *β*-propeller motif	Not mentioned	China
			c.1619T>C	p.L540P	Second epidermal growth like factor	Not mentioned	

Seo et al. [59]	51	4	c.731C>G	p.T244R	First *β*-propeller motif	3A/2B stage FEVR	Korea
			c.1330C>T	p.R444C	Second *β*-propeller motif	2A stage FEVR	
			c.1833dupG	p.C612VfsX25	Premature termination	1B/4A stage FEVR	
			c.4098C>G	p.D1366E	Low-density-lipoprotein receptor-like ligand binding domains	3B stage FEVR	

Zhang et al. [85]	4	4	c.C1042T	p.R348W	First epidermal growth-like factor	Not mentioned	China
			c.G1141A	p.D381N	Second *β*-propeller motif	Not mentioned	
			c.C1870T	p.R624W	Second epidermal growth-like factor	Not mentioned	
			c.A4550G	p.Y1517C	Cytoplasmic tail	Not mentioned	

Tang et al. [31]	100	10	c.1058G>A	p.R353Q	First epidermal growth-like factor	Ilateral retrolenticular fibrotic mass and total retinal detachment	China
			c.1183C>T	p.R395W	Second *β*-propeller motif	Falciform retinal fold	
			c.1318A>T	p.I440F	Second *β*-propeller motif	Retinal fold	
			c.1582G>A	p.E528K	Second *β*-propeller motif	Peripheral vascular deficiencies	
			c.1942G>A	p.V648I	Second epidermal growth-like factor	Rhegmatogenous retinal detachment	
			c.2738G>T	p.C913F	Third epidermal growth-like factor	Retinal fold and macular dragging	
			c.4087G>C	p.D1363H	Low-density-lipoprotein receptor-like ligand binding domains	Falciform retinal fold	
			c.4733C>T	p.T1578M	Cytoplasmic tail	Retinal fold	
			c.92-2A>C	Splice site mutation	Premature termination	Ilateral retrolenticular fibrotic mass and total retinal detachment	
			c.4488 + 2T>G	Splice site mutation	Premature termination	Retinal folds	

Rao et al. [29]	31	5	c.4205G>A	p.G1402D	Transmembrane domain	Falciform fold	China
			c.2237G>C	p.R746P	Third *β*-propeller motif	Peripheral avascular zone	
			c.2618A>T	p.K873M	Third *β*-propeller motif	Peripheral avascular zone	
			c.1384C>T	p.R462X	Second *β*-propeller motif	Complete retinal detachment	
			c.2817_2827+1del12bp	p.N940fs	Premature termination	Complete retinal detachment	

Liu et al. [83]	10	5	c.542T>G	p.M181R	First *β*-propeller motif	Diagnosed with FEVR, symptoms not mentioned	China
			c.1197G>T	p.R399S	Second *β*-propeller motif	Diagnosed with FEVR, symptoms not mentioned	
			c.1481G>A	p.R494Q	Second *β*-propeller motif	Diagnosed with FEVR, symptoms not mentioned	
			c.1507G>A	p.G503R	Second *β*-propeller motif	Diagnosed with FEVR, symptoms not mentioned	
			c.2626G>A	p.G876S	Third epidermal growth-like factor	Diagnosed with FEVR, symptoms not mentioned	

Pefkianaki et al. [84]	1	1	c.2234C>T	p.A745V	Third *β*-propeller motif	Extensive exudative retinopathy and shallow retinal detachment	USA

**Table 4:** Spectrum of *TSPAN12* gene mutations among patients with familial exudative vitreoretinopathy.

Studies	No. of patients	No. of mutations	DNA variant	Coding effect	Location of the amino residue	Mutant phenotypes	Country of origin
Savarese et al. [94]	1	1	c.668T>C	p.L223P	Transmembrane domain	No sign of neovascularization	Pakistan

Poulter et al. [93]	58	5	c.67-1G>C	p.L23GfsX66	Transmembrane domain, premature termination	Bilateral retinal folds	Mexican and Pakistan
			c.146C>T	p.T49M	First extracellular loop	Bilateral congenital cataract, large retinal fold	
			c.285 + 1g>a	p.R50DfsX12	Premature termination	Bilateral congenital cataract, large retinal fold	
			c.413A>G	p.Y138C	Second extracellular loop	Peripheral retina avascularity	
			c.668T>C	p.L223P	Transmembrane domain	Bilateral retinal folds, funnel retinal detachments	

Poulter et al. [6]	70	7	c.68T>G	p.L23X	Transmembrane domain	Bilateral retinal folds and unilateral, persistent hyperplastic primary vitreous	USA, UK, Britain, Japan, Australia
			c.149 + 3a>g	Splice-site mutation	Premature termination	Unilateral retinal fold	
			c.218_219insGCTGTTT	p.F73LfsX119	Premature termination	Macula ectopia, with a large retinal fold	
			c.302T>A	p.L101H	Transmembrane domain	Lassic signs of FEVR	
			c.361-5_361-1delaccag	Splice-site mutation	Premature termination	Bilateral temporal retinal avascularity	
			c.419T>A	p.L140X	Second extracellular loop	Bilateral retinal folds	
			c.629T>G	p.M210R	Bilateral macular traction	Bilateral macular traction	

Nikopoulos et al. [68]	43	2	c.709G>C	p.A237P	Transmembrane domain	Avascular peripheral retina	Netherlands
			c.562G>C	p.G188R	Second extracellular loop	Avascular peripheral retina	

Yang et al. [96]	49	3	c.146C>T	p.T49M	First extracellular loop, conserved residue	Falciform retinal folds	China
			c.313T>C	p.C105R	Transmembrane domain, conserved residue	Midperipheral retina, an avascular zone on the peripheral retina	
			c.601delC	p.L201FfsX14	Conserved residue	Inferotemporal dragging of the optic disc and macula	

Gal et al. [91]	64	1	c.542G>T	p.C181F	Second extracellular loop, form disulfide bonds	Bilateral visual impairment, various ocular abnormalities	Israel
Xu et al. [95]	85	3	c.177delC	p.Y59fsX67	Premature termination	Falciform retinal folds	China
			c.C254T	p.T85M	Intracellular loop	Pigment deposit, dragged disc	
			c.566G>A	p.C189Y	Second extracellular loop, form disulfide bonds	Bilateral retinal folds	

Kondo et al. [92]	90	2	c.419T>A	p.L140X	Second extracellular loop	Abnormal retinal vessels with vitreous degeneration	Japan
			c.734T>C	p.L245P	C-terminal cytoplasmic tail	Retinal fold resulting	

Seo et al. [59]	51	1	c.56T>G	p.L19R	Transmembrane domain	3A stage FEVR	Korea

Ganeswara Rao Musada et al. [2016]	110	3	c.125T>C	p.V42A	First extracellular loop	Diagnosed with FEVR, symptoms not mentioned	India
			c.334G>A	p.V112I	Second extracellular loop	Diagnosed with FEVR, symptoms not mentioned	
			c.479G>A	p.C160Y	Second extracellular loop	Diagnosed with FEVR, symptoms not mentioned	

Tang et al. [31]	100	8	c.2T>C	p.M1T	N-terminal domain	Not mentioned	China
			c.464G>C	p.R155T	Second extracellular loop	Not mentioned	
			c.438-439insT	p.T147YfsX12	Premature termination	Total retinal detachment and massive vitreous proliferation	
			c.655delC	p.Q219NfsX5	Premature termination	Total retinal detachment	
			c.916-918 + 3delTAAAAA	p.∗306Eext∗35	Elongated protein	Peripheral avascular retina	
			c.150-1G>A	Splice acceptor mutations	Not applicated	Not mentioned	
			c.285 + 1G>A	Splice acceptor mutations	Not applicated	Not mentioned	
			c.469-1G>A	Splice acceptor mutations	Not applicated	Not mentioned	

Iarossi et al. [24]	8	1	c.67-2A>G	Defective splicing	Not applicated	Falciform retinal fold	Italia

Rao et al. [29]	31	1	c.345T>G	p.Y115X	Second extracellular loop	Falciform folds, complete retinal detachment	China

Liu et al. [83]	10	1	c.566G>A	p.C189Y	Second extracellular loop		China

Schatz and Khan [58]	3	1	c.565T>C	p.C189R	Second extracellular loop, affects cystine residues forming	Total retinal detachment	Sweden
